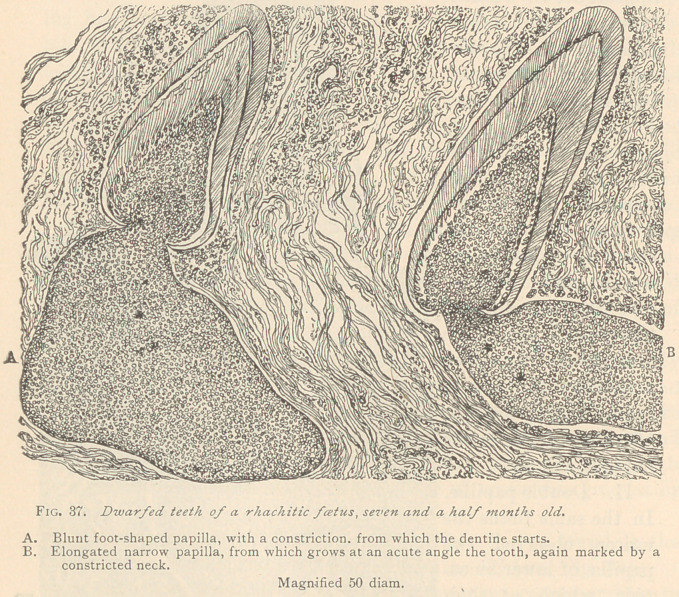# Contributions to the History of Development of the Teeth

**Published:** 1887-11

**Authors:** Carl Heitzmann, C. F. W. Bödecker


### Independent Practitioner.

VOL. VIII. NOVEMBER, 1887. NO. 11.

NOTE.—No paper published or to be published in another journal will be accepted for this department. All papers must be in the hands of the Editor before the first day of the month preceding that in which they are expected to appear. Extra copies will be furnished to each contributor of an accepted original article, and reprints, in pamphlet form, may be had at the cost of the paper, press-work and binding, if ordered when the manuscript is forwarded. The Editor and Publishers are not responsible for the opinions expressed by contributors. The journal is issued promptly, on the first day of each month.

### Original Communications.

### CONTRIBUTIONS TO THE HISTORY OF DEVELOPMENT OF THE TEETH.

BY CARL HEITZMANN, M. D., AND C. F. W. BÖDECKER, D. D. S., M. D. S.

CONTINUED FROM PAGE 577.

### V. THE DEVELOPMENT OF TEETH IN EMBRYOS AFFECTED WITH RHACHITIS.

Among the numerous embryos, the teeth of which the writers have examined, there were three affected with so-called congenital rhachitis. One of these was a seven-months fœtus, another seven months and a half, while the third was eight months old. Macroscopically the two former did not exhibit any symptoms of rhachitis, and it was only upon microscopical examination of the jaw bones that we were enabled to recognize the morbid process, whose characteristic feature is a new formation of hyaline cartilage in the place of bone tissue. The trabeculæ of the cancellous bone tissue were scantier, and the bone corpuscles within them were much larger than those in normal bone tissue. In many places, however, hyaline cartilage was present instead of bony trabeculæ, and in the medullary spaces between them. The cartilage corpuscles often were of a brown color, caused either by previous hemorrhage or by a new formation of blood corpuscles and hæmoglobine. The third fœtus, which was eight months old, showed the symptoms of congenital rhachitis in such a degree that all bone tissue throughout the body was lacking, and only in the lower jaw were found a few trabeculæ. This fœtus was delivered by a healthy woman, who during her pregnancy, for a number of months fed dogs, cats and rabbits with soup, meat, bread and vegetables respectively, mixed with lactic acid, for the purpose of artificially producing rhachitis and osteomalacia in these animals. These experiments were made by Carl Heitzmann (see Microscopical Morphology, 1883) and proved to be successful, since dogs and cats, if treated with lactic acid soon after birth, became rhachitic, and if treated with lactic acid for several months in succession, became affected with osteomalacia.

The woman who gave birth to this fœtus was healthy during pregnancy, and remained so after delivery. The fœtus, on the contrary, died shortly after birth, from intra-cranial hemorrhage caused by pressure during labor, on account of the complete absence of cranial bones.

The abnormal occurrences in the teeth of these three rickety embryos were so striking and so numerous that we made an attempt to arrange them under a number of headings, being aware of the fact that different chronic ailments of both mother and fœtus, more especially rhachitis of the fœtus, reflect on the growth of the teeth, and leave marks in the shape of transverse grooves or furrows upon them.

I. Premature eruption of illy developed teeth was found in the lower jaw of a rhachitic fœtus, seven months old (see Fig. 36).

This tooth had reached a stage of development corresponding to about the seventh month of intra-uterine life, but had grown above the level of the gum, and was plainly visible to the naked eye. This specimen helped to settle a mooted question concerning the origin of Nasmyth's membrane, the cuticle of the tooth. The writers observed that the flat epithelial layer covering the summit, and the dentine at the deeper portion of the tooth, is a direct reduplication of the flat epithelial layer of the gum, as mentioned in a former article (see *Dental Cosmos,* 1878 and 1879). The papilla is moderately supplied with blood vessels, and presents a markedly myxomatous structure, especially at its lower part, which appears lobulated. It is bordered by a structureless layer at its lower portion, which we consider a normal feature, and which is always present previous to the appearance of odontoblasts. The gum is composed of a loose, delicate, fibrous connective tissue. At the right side of the specimen, near the lower portion of the papilla, there appears an isolated layer of myxomatous tissue, which extends below the papilla, bearing the characteristics of the enamel organ. Its outer periphery shows a number of buds or nests, which are the offspring of the external epithelium.

FIG. 36. *Premature eruption of a lower incisor of a rhachitic fœtus, seven months old.*

G. Gum.

C. Fibrous connective tissue.

E. O. Myxomatous enamel organ.

Magnified 50 diam.

II. Double papillæ. In the same fœtus the writers observed two papillæ of lower bicuspids, which at their bases were united into one continuous broad mass, whereas higher up there was seen a cancellous bony structure, separating the papillæ, which proves that they belonged to two separate teeth. The structure of these papillæ was markedly myxomatous, similar in appearance to that of the papillæ of pig's teeth. The enamel organ appeared to be in full devolopment, being lined by the internal epithelium, which was broken up into medullary tissue, and an external epithelium broken up into epithelial nests. Along the internal epithelium peculiar folds and indentations are visible, which the writers propose to describe later on. No trace of dentine or enamel was visible upon these germs.

A

B

FIG. 37. *Dwarfed teeth of a rhachitic fœtus, seven and a half months old.*

A. Blunt foot-shaped papilla, with a constriction. from which the dentine starts.

B. Elongated narrow papilla, from which grows at an acute angle the tooth, again marked by a constricted neck.

Magnified 50 diam.

III. Dwarf teeth. In the rhachitic fœtus which was seven and a half months old, the writers met with minute teeth which were in a stage of development corresponding to the age of the fœtus (See Fig..37.)

The papillæ are of very irregular shape, being partly blunt, partly elongated, and sharply marked by a constriction at the place corresponding to the neck of the future tooth. Above the neck the papillæ are of the usual lancet or myrtle-leaf shape, exhibiting along their borders either odontoblasts or rows of medullary corpuscles, and showing comparatively few blood vessels. The dentine is curved around the constriction in a marked degree, surrounding the neck of the tooth like a cap. Its canaliculi are wide but without pathological features. The enamel is likewise to bė considered normal, but the enamel organ is missing in all of these specimens, which indicates that the myxomatous reticulum was exhausted. The external epithelium only shows its presence by small epithelial nests and buds along the already formed enamel.

(TO BE CONTINUED.)

## Figures and Tables

**Fig. 36. f1:**
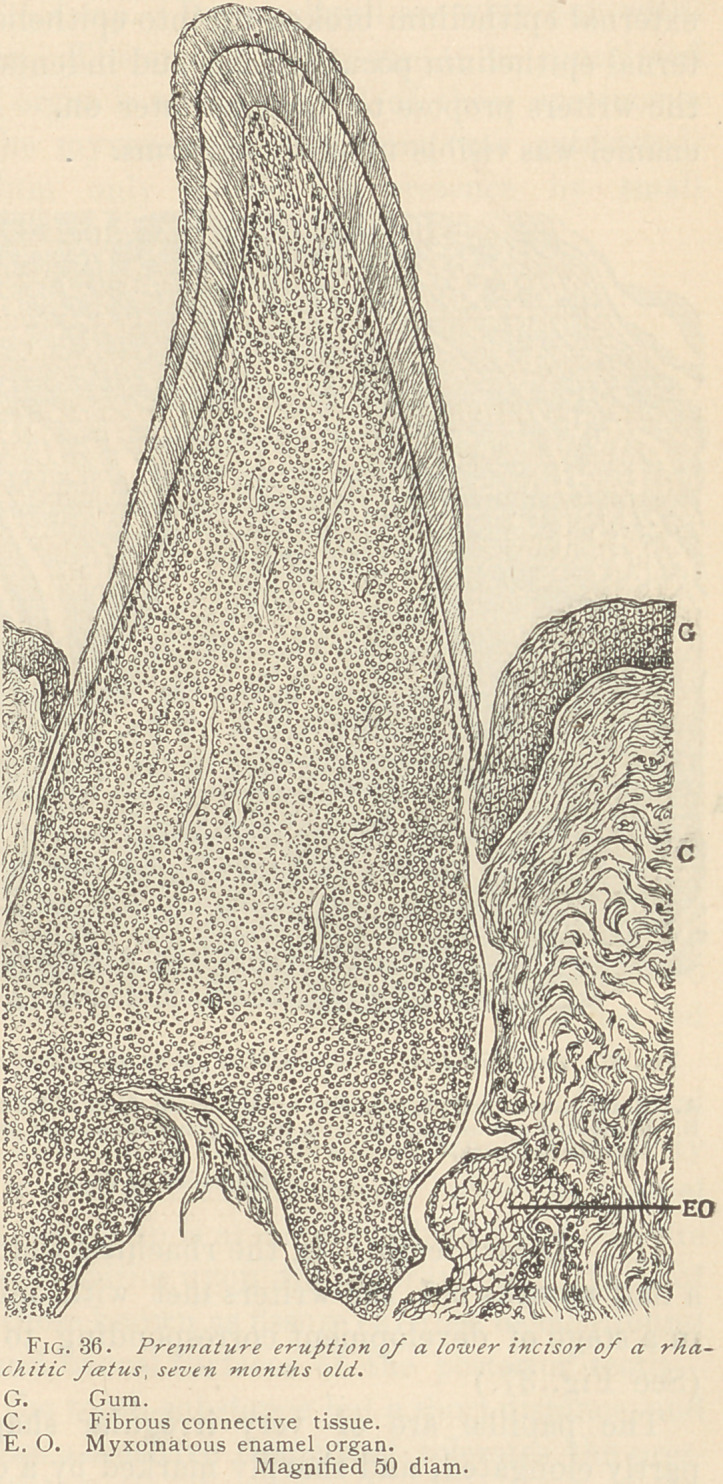


**Fig. 37. f2:**